# Analysis of Selected Properties of Microporous PLA as a Result of Abiotic Degradation

**DOI:** 10.3390/ma15093133

**Published:** 2022-04-26

**Authors:** Aneta Tor-Świątek, Tomasz Garbacz, Petr Stloukal

**Affiliations:** 1 Faculty of Mechanical Engineering, Lublin University of Technology, 36 Nadbystrzycka, Str., 20-816 Lublin, Poland; a.tor@pollub.pl; 2 Centre of Polymer Systems, Tomas Bata University in Zlin, Tř. T. Bati 5678, 760 01 Zlin, Czech Republic; stloukal@utb.cz

**Keywords:** polylactide, blowing agent, microporous extrusion process, thermal behavior

## Abstract

In the study, an investigation was made into the hydrolytic degradation behavior of the microporous polylactide (PLA) in the initial stage in three biological buffer solutions with various pH-simulating body fluids in comparison with pure PLA. Studies also include the analysis of selected mechanical properties and physical structures. A microporous PLA was obtained by melt extrusion using a chemical blowing agent. The rate of Mw decrease induced by hydrolysis over 35 days of microporous PLA was roughly comparable to the pure material. The rate of depolymerization was slightly accelerated at an acid pH due to acid-catalyzed hydrolysis at the end of the observed period. The mechanical analysis showed the influence of various pH on the obtained results.

## 1. Introduction

Over the years, the world market for polymers has evolved towards the increased use of environmentally friendly materials. The high interest of manufacturers in natural and biodegradable materials results from their willingness to meet the needs of the ever-increasing group of consumers who value the “Zero Waste” principle. At the head of this group of materials, which attracted the above-mentioned attention of manufacturers, is polylactide (PLA), which is the most famous biodegradable material in the world and should be undoubtedly mentioned. Despite its many advantages, PLA is a relatively expensive material, which has an impact on its applicability. PLA is a material that can successfully replace other commonly used plastics such as polypropylene (PP) or polystyrene (PS) in packaging applications [1,2,3,4,5,6,7,8], automotive [9,10], consumer goods, electronics [11,12], 3D printing applications [13,14,15,16,17], and biomedical devices [18,19].

In order to reduce the manufacturing costs and improve the selected mechanical and physicochemical properties, modifications of polylactide are applied, and its biodiversity is maintained. The most frequently applied modifications include mixtures with other polymers, such as polyethylene terephthalate (PET), polybutylene terephthalate (PBT), or the increasingly used poly (butylene adipale-co-terephthalat (PBAT) [20,21,22,23,24,25,26]. Often, research related to the composition of PLA with PA (polyamides) and its different types and applications is undertaken [27,28,29].

Numerous studies on the various property changes of the modified PLA materials with powder and fiber additives are also highly interesting [30,31,32,33,34]. Powder additives, such as talc [35,36] and basalt [37,38], are commonly used in research. Nevertheless, more often than not, organic fiber additives are applicable. Its usage depends on its availability in the particular country and region where that type of fiber waste is produced. The research was conducted to determine the suitability of these types of fillers for modification of PLA concerns, and, among others, sisal [39,40], jute [41], kenal [42], cotton [43], bamboo [44,45], and, most of all, flax [46,47,48,49,50].

The changes in the properties of the modified PLA composition may also relate to the reduction of its weight, which can be obtained as a result of the porous and microporous processes. The PLAs porous process is aimed at obtaining products with a reduced density, and free from dents on the surface, showing minimal shrinkage. Appropriately selected materials and processing conditions make it possible to obtain composites with new physical and technological properties. This also applies to the visual alteration of the obtained surfaces made of the product’s composition. One of the possibilities for obtaining a porous structure in materials is the use of chemical or physical blowing agents [51,52,53]. This type of porous material consists of introducing a blowing agent into the material, which decomposes under appropriate conditions, in particular, pressure and temperature, and gives off gas. This gas is dissolved in plastic. Then, after reducing the pressure, blowing agent begins to separate from the material, creating a porous structure that should be fixed by solidifying the material and cooling the cellular product [54,55].

In recent years, microporous PLA has attracted great attention in the medical area due to its adequate mechanical properties, good biocompatibility, and biodegradability [44,49,56]. Several advantages of PLA production should be highlighted. Microporous PLA is renewable, as is its production, due to the high quantity of carbon dioxide being consumed, thus providing a significant energy saving process. Furthermore, it is recyclable and compostable, and its physical and mechanical properties can be partially customized by properly selecting the polymer architecture [57,58,59,60,61].

Regarding conventional polymers such as PE, PP, and PVC, the production of their microporous forms with various commercial blowing agents by melt extrusion or injection molding has been intensively studied in recent decades [51,52,53,55,62,63]. However, studies concerning the investigation of the microporous extrusion of PLA have appeared only in relatively recent times [64,65,66,67]. Some of them deal with the microporous extrusion of PLA using supercritical CO_2_ [68,69]. Several previously published works have also focused on microporous PLA composites and their mixtures with other polymers [70,71,72,73,74].

The main objective of these studies was to produce a microporous material with controllable cell morphology and mechanical properties. However, there is a significant lack of information concerning the effect of the porous structure on the susceptibility to hydrolysis in various environmental conditions. From the viewpoint of medical applications, the kinetics of degradation (bioresorption) of implant material is considered one of the key factors [75,76,77,78,79,80]. Therefore, the aim herein was to investigate the hydrolytic behavior of microporous PLA modified with a commercial blowing agent in three biological buffer solutions with various pH-simulating body fluids.

## 2. Materials and Methods

Polylactide of resin type 2002D was obtained from NatureWorks^®^ Ingeo™, (Blair, NE) USA, Czech Republic. The blowing agent Hydrocerol 530 was purchased from Clariant Masterbatch GmbH & Co., OHG, Ahrensburg, Germany. The blowing agent was dosed into PLA in the amount of 1% of the mass in relation to the mass of the pure PLA.

**Figure 1:**
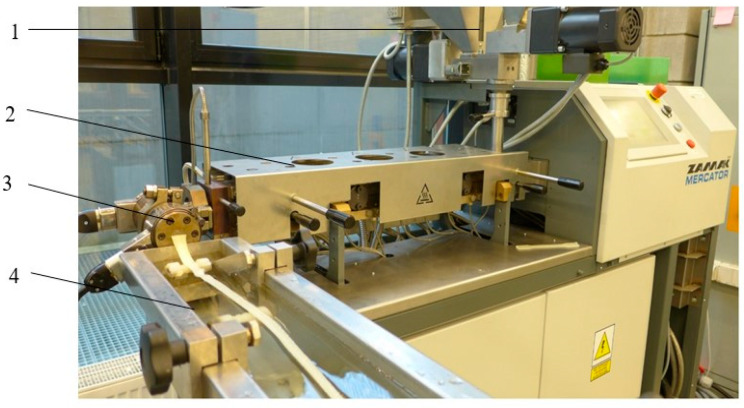
Twin-screw extruder machine: 1—dosing device, 2—plasticizing system, 3—extruder head, 4—cooling device.

Prior to the film extrusion process, PLA pellets and Hydrocerol 530 were pre-dried at 40 °C for 24 h. Then, microporous extrusion process of samples was run with the use of the twin-screw extruder machine EHP 2 × 20 Sline (Figure 1), produced by Zamak Mercator (Skawina, Poland). The extruder’s plasticizing unit screw had an L/D ratio of 25 and an outside diameter, D, of 20 mm. The rotational speed of the extruder screw ranged from 0 to 200 rpm and was adjusted continuously by 50 rpm. The technological line also consisted of a head for profile extrusion. The head had a replaceable extruder die to enable extrusion of profiles with different sizes and shapes, both symmetric and asymmetric.

**Figure 2:**
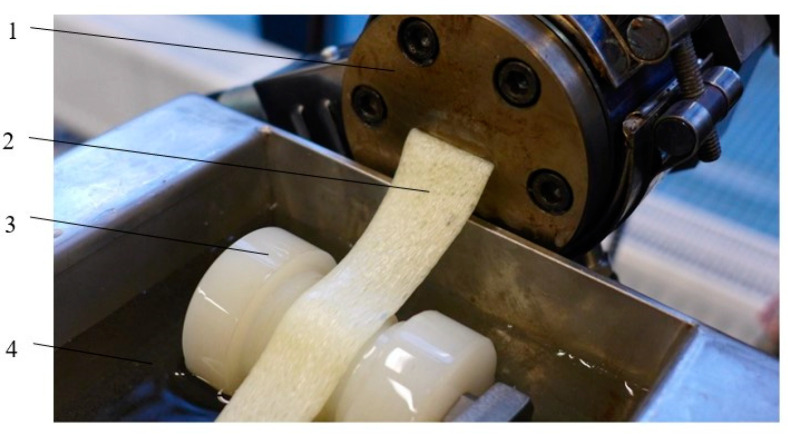
View of the extrusion head with the extruded tape with microporous PLA: 1—extruder head, 2—extrudate, 3—guide rollers, 4—cooling factor (water).

Used for tape profile extrusion, the extruder die had a width of 20 mm and a height of 3.0 mm (Figure 2). The extruder head was made up of two heating zones and two corresponding ring-shaped heaters mounted on the head body. The extrusion line also consisted of a cooling device that had a length of 1740 mm, width of 220 mm, and depth of 200 mm. The cooling factor used was water, with a temperature of 14 °C. In the tests, was also used a belt haul-off; the belt had a width of 100 mm and a length of 2000 mm.

The process was conducted at the temperature of the heating zones in the plasticizing unit set to 180, 170, 170, 170, 165, 165, 165, 150, and 125 °C, respectively, while the temperature of the extrusion head in the heating zones was set to 180 °C. The mechanism of producing microporous PLA consisted in introducing a mixture of plastic granules and blowing agents into the plasticizing system of the extruder. After reaching the temperature of 170 °C, the blowing agent decomposed with the release of the active substance (a mixture of appropriately proportioned chemical compounds such as azodicarbonamide), causing a change from a solid to a porous structure.

The obtained samples were subjected to hydrolytic degradation in three biological buffers. Buffer pH 2.4 simulates pH of stomach acid in lower part of stomach, pH 7.4 simulates pH of saliva, and pH 12 simulates pH of intestines.

Scanning electron microscopy (Phenom Pro, prod. TESCAN, Brno, Czech Republic) was carried out to observe the porosity of microcellular PLA matrix after the blowing process. The microscope was operated in high-vacuum mode at an acceleration voltage of 10 kV.

HT-GPC 202 chromatographic system (prod. Agilent, Santa Clara, CA, USA) has been used for GPC analysis. Equipped with a dual detection setup, allowing refractive index and viscometric response detectors. Overnight THF (3 mg/mL) was used as a dissolvent for the examiner samples. Separation and detection were in place on PL gel-mixed bed columns in the following proportion 1 × Mixed-A, 300 × 7.8 mm, 15 μm particles + 1 × Mixed-B, 300 × 7.8 mm, 10 μm particles + 1 × Mixed-D, 300 × 7.8 mm, 5 μm particles. With 40 °C in THF the flow rate equals 1.0 mL/min and injection volume at 100 μL. Universal calibration with narrow polystyrene with standards ranging from 580 to 271,000 g/mol (Polymer Laboratories Ltd., Church Stretton, UK) has been performed on GPC system of the tested samples. The average weight (Mw), number (Mn), and molar mass dispersity (Đ = Mw/Mn) of the tested material have been determined from peaks corresponding to the relevant polymer fraction. All data processing delivered via Cirrus software, Marlow, UK.

To determine thermal properties DSC on a DSC1 STAR System (Mettler Toledo, Greinfensee, Switzerland) has been employed. The samples of a film weighing ca 5 mg were deposited in aluminum pans. With nitrogen flow set at 50 mL/min and a corresponding heating setting adjusted initially with heating cycle ranging from 0 to 200 °C (10 °C/min) and sustaining the same level for 2 min, then cooling to 0 °C (10 °C/min). The temperature of 0 °C has been kept for 2 min, then extending heating scan to 200 °C. Achieving melting point and exothermal response in relation to cold crystallization (Tc) in the first heating cycle. The area of glass-transition temperature (Tg) obtained in the second heating scan. Established by the following Equation (1) [23] the degree of crystallinity *χ_c_*:

(1) $$\chi_{c}=\left(\frac{\Delta H_{m}-\Delta H_{c}}{\Delta H_{m}^{0}}\right)\times 100\%$$

where, Δ*H_m_* is the heat of fusion, Δ*H_c_* represents cold crystallization enthalpy and $\Delta H_{m}^{0}$ is the tabulated heat of fusion for theoretically 100% crystalline PLA homopolymer (93.1 J·g^−1^) [23]. 

Tensility can be easily obtained with the use of a universal test device, such as the M350-5 CT Materials Testing Machine (Testometric Company, Lancashire, UK) in accordance with PN EN ISO 527-1-4 at a crosshead speed of 5 mm/min. Tensile test samples were cut from compression mold plates with the following dimensions 60 × 4.0 × 0.1 mm. The samples were stored in regular laboratory conditions for 24 h prior to the testing. A minimum of eight specimens from each group were tested.

## 3. Results

**Figure 3:**
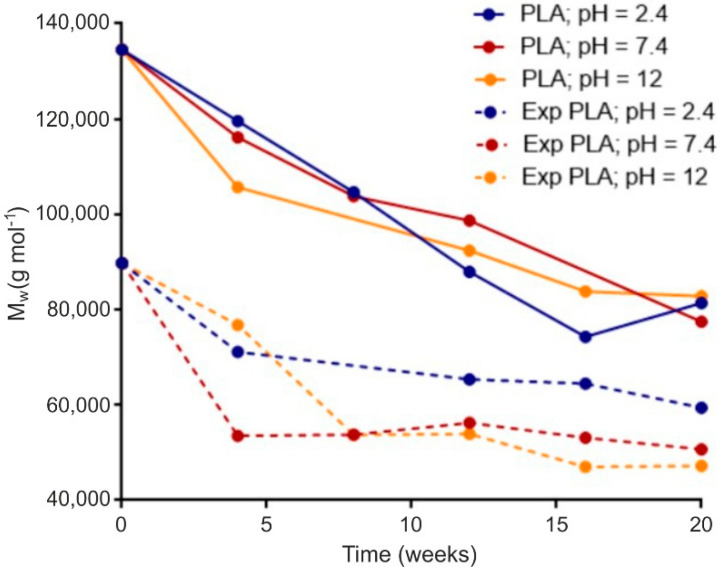
Molecular weight evolution for materials of PLA and expanded PLA in various phosphate buffer during abiotic hydrolysis at 37 °C.

**Figure 4:**
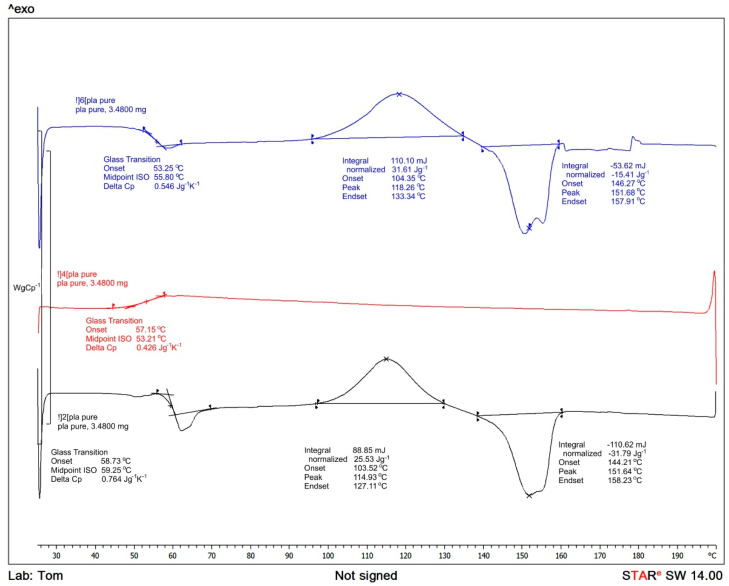
Thermal curves of compact PLA.

**Figure 5:**
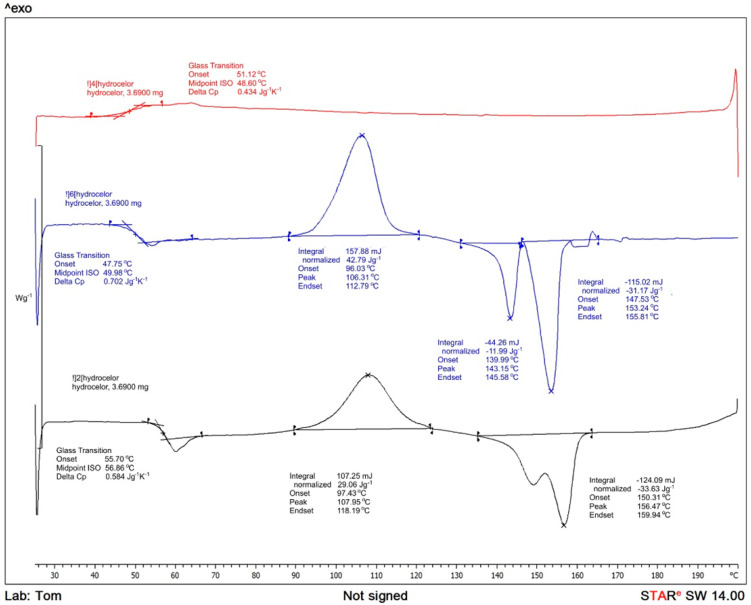
Thermal curves of expanded microporous PLA with blowing agent Hydrocerol 530.

**Figure 6:**
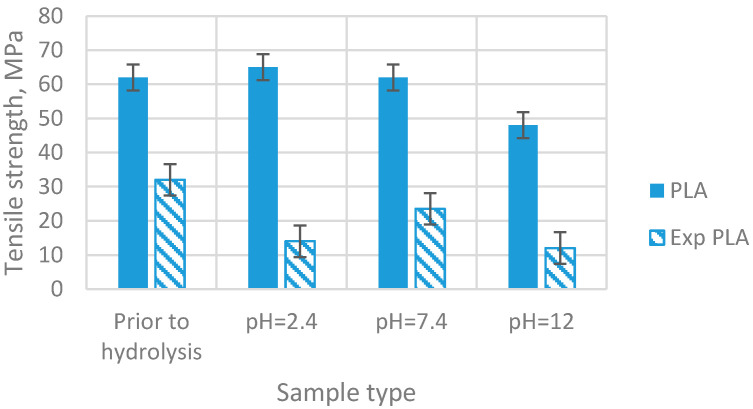
Tensile strength of samples prior to and after exposition to abiotic hydrolysis in various pH buffer solutions (immersion time—20 weeks).

**Figure 7:**
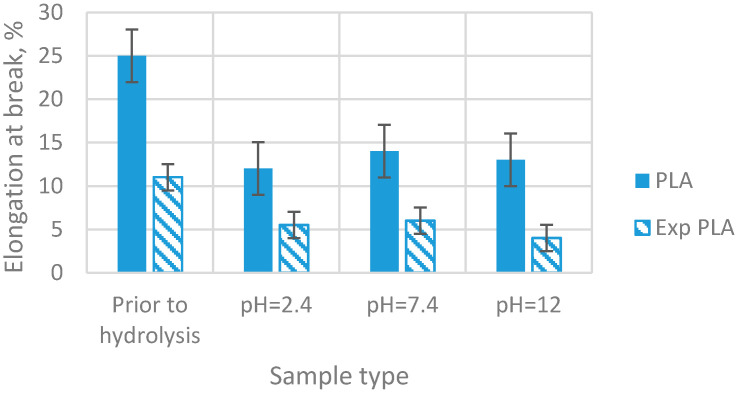
Elongation at break of samples prior to and after exposition to abiotic hydrolysis in various pH buffer solutions (immersion time—20 weeks).

**Figure 8:**
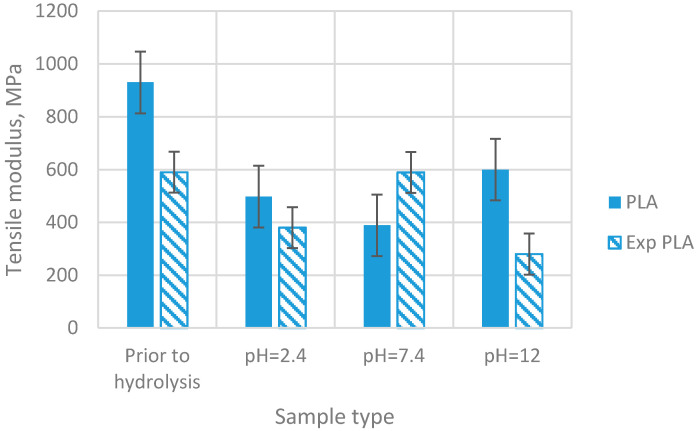
Tensile modulus of samples prior to and after exposition to abiotic hydrolysis in various pH buffer solutions (immersion time—20 weeks).

**Figure 9:**
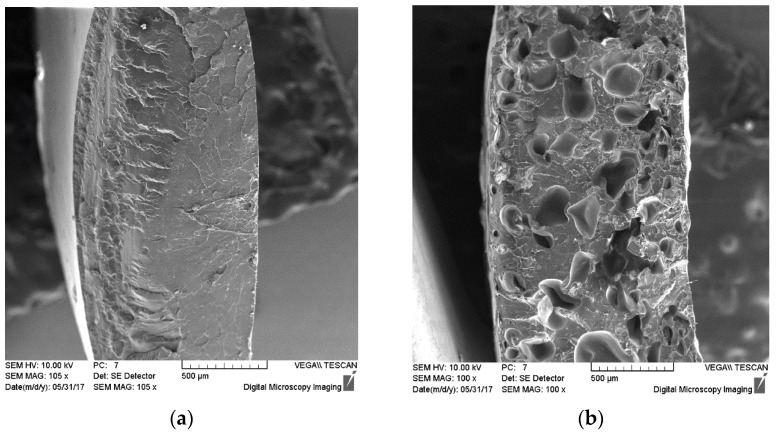
Scanning electron micrographs of pure compact PLA (**a**) and microporous PLA (**b**).

Following the conducted tests, results of the analyses of the selected properties of samples prior to degradation (Table 1) were obtained. In addition, results of the impact of abiotic hydrolysis on the selected physicochemical (Figure 3) thermal (Figure 4 and Figure 5 and Table 2), mechanical (Figure 6, Figure 7 and Figure 8), and structural (Figure 9) properties of PLA modified with a blowing agent were recorded.

The investigations into the selected properties prior to degradation (Table 1) showed a reduction in the molecular weight (Mw) by 1.5% for PLA after processing and by a subsequent 44% after adding the blowing agent.

A slight increase in the molar-mass dispersity for the pure PLA samples after processing with the porous extrusion method was observed. The addition of the blowing agent resulted in a decrease in the crystallization temperature (Tc) by 6%, the glass-transition temperature (Tg) by approximately 2%, the total enthalpy of fusion by 131%, and an increase in the enthalpy of crystallization by 184%.

The research (Table 2) has revealed that the increased degradation time reduces the glass-transition temperature (Tg), both in samples with pure and with microcellular PLA (Exp PLA). This change is not affected by the pH of the sample. Moreover, degradation time resulted in a decrease in the melting point (Tm) of the pure PLA and an increase for the microporous samples (Exp PLA).

The addition of the blowing agent slightly increased the melting point (Tm) for degradation times of 12 and 20 weeks, as well as pH 7.4 and 12 of the sample. This increase amounts to 3% on average. In the case of the crystallization temperature (Tc), a decrease in the tested value, both due to the degradation time and the addition of the blowing agent, was observed. For individual pH values, this decrease amounts to 5% on average in pure PLA (for pH 2.4, time 0 weeks) and 9% in microporous (Exp PLA) (for pH 2.4, time 20 weeks).

The value of the total enthalpy of fusion has decreased for the pure PLA by 30% on average with an increase in degradation time; for the microporous PLA (Exp PLA) by 3.5% on average. The value of the crystallization enthalpy increased for the pure PLA by 40% on average; for the microporous (Exp PLA) by 2.5% on average.

## 4. Discussion

Figure 3 displays the evolution of the molecular weight of neat PLA and microporous PLA (Exp. PLA) over the first 35 days of abiotic hydrolysis in three different pH buffer solutions. A slight diminishment in weight average molecular weight (Mw) was detected in microporous PLA (Exp. PLA) after preparation, unlike in the pure PLA, as a consequence of the zinc compound contained in the blowing agent, which is known as an effective catalyzer of PLA degradation. Comparing both materials, the rate of Mw decrease induced by hydrolysis over 35 days was comparable, suggesting that the porous structure had no significant impact on susceptibility to hydrolysis at the beginning of the process in our case. As was expected, the chain scission of the ester bond in PLA was slightly accelerated by acid pH due to acid-catalyzed hydrolysis in both samples at the end of the observed period.

Based on the results of the thermal analysis (Figure 4 and Figure 5), it can be concluded that during the first heating cycle, the glass-transition temperature of the microporous PLA (Exp. PLA) is lower in comparison to the temperature of the pure PLA and equals 49.98 °C. Not to mention that the maximum peak for the DSC curve corresponding to the cold crystallization for the sample modified with the blowing agent is lower (106.31 °C) in relation to the pure PLA sample (118.26 °C)—first heating cycle. Moreover, on the DSC curve for microporous PLA, a double melting peak in the range from 130 °C to 200 °C is detected—the first heating cycle. This is most likely due to the evaporation of the volatile components of the blowing agent, Hydrocerol 530. The observed endothermic effect in this temperature range is reversible—it is visible also during the second heating cycle (−33.63 J/g), which may indicate the melting of the polyester thermoplastic groups contained in PLA. The article [72] demonstrates that the degree of crystallinity in the PLA/PHBV composite remains fairly constant regardless of the amount of PLA in the composite. Moreover, samples containing 50% or more of PHBV exhibit two endothermic peaks at approximately 156 and 145 °C, which correspond to common crystal perfection behavior seen in many semi-crystalline polymers. Similar results are observed for pure PLA. The glass transition was observed at 60C and there was no melting or crystallization peak observed, which indicates the sample to be fully amorphous under the DSC experimental conditions.

The analysis of the mechanical properties revealed a slight and gradual decrease in tensile strength (Figure 6) with the change in the pH of PLA. The highest decrease in tensile strength of the pure PLA was observed in the basic medium (pH 12), and it amounts to 48 MPa. For the material that is modified with the blowing agent (exp PLA), these changes are not steady. In an acidic medium (pH 2.4), the strength value for exp PLA is lower by approximately 100% in comparison to the pure PLA. In a neutral medium (pH 7.4), a slight increase in strength of approximately 80% is observed. Then, a decrease in this value of approximately 100% at pH 12 is detected.

The elongation at break (Figure 7) decreases significantly after hydrolysis of the pure PLA by 52%, then it increases slightly. The results of elongation at break are the same for the modified PLA.

In the case of the elastic modulus (Figure 8), after the first hydrolysis of PLA, a decrease of 46% is recorded. Following the second hydrolysis, the modulus decreases by 21%, and then it increases by approximately 54%. The same situation is observed in exp PLA for pH 2.4 and 7.4. For pH 12, the value of elastic modulus remains at the same level as for pH 7.4.

A different nature of the changes is presented in [64], where the tensile modulus of PLA foams decreased with increasing saturation pressure, but the specific tensile modulus of PLA foams was still 15–80% higher than that of solid PLA. In the cited article, tensile strength and elongation at break first increased with increasing saturation pressure up to 16 MPa and then decreased with further increasing saturation pressure (20 MPa and 24 MPa) at which an open-cell structure was produced. Moreover, in [47], it was found that a reduction of fiber lengths took place during the extrusion process and that the addition of flax fibers to the PLA/PCL blend resulted in an increase in elastic modulus and biodegradation rate. The composite impact strength was significantly improved at a 30 wt% PCL fraction.

It is clear from the data received that after the exposition of samples to hydrolysis, the best average results of mechanical properties of the pure PLA samples were obtained in neutral and basic medium, and for exp PLA samples in neutral medium.

The SEM micrographs (Figure 9) show the freeze-fracture surfaces of neat PLA (a) and microporous PLA (b). Microporous PLA exhibits a closed-porous structure with non-homogenous size pores in comparison with non-porous pure PLA. A similar morphology of the structure was illustrated in articles [67,68], where closed pores were obtained with cell densities on the order of 109 cells/cm^3^ and cell sizes of around 10 μm, and melt strength governs cell morphology, with cell density, closed-cell content, and expansion ratio increasing as a function of both molecular weight and branching density.

## 5. Conclusions

The thermal stability of microporous PLA during melt extrusion was shown to be slightly weaker due to the acceleration of degradation by the zinc compound contained in the blowing agent in comparison with pure PLA. However, the susceptibility to depolymerization induced by hydrolysis over 35 days in various pH buffer solutions was comparable for both materials.

The investigation into the mechanical properties revealed the impact of all variable factors, i.e., the addition of the blowing agent, pH, and degradation time. The highest drop in the tested values for the samples modified with the blowing agent was observed in the acidic medium, and the lowest—was in the neutral medium. It was also found that the addition of the blowing agent to PLA changed the characteristics of the DCS curve and had an impact on the values of a particular reference temperature of tested materials. It is, therefore, necessary to extend the scope of research to include the increased dosing of the blowing agent in the amount of 1.5–2% of the mass.

## Figures and Tables

**Table 1:** Selected material properties of samples after preparation and before degradation experiments.

Sample	M_w_^a^ [kg·mol^−1^]	Đ ^b^	T_m_1_ ^c^ [°C]	ΔH_m_1_ ^d^ [J·g^−1^]	T_m_2_ ^e^ [°C]	ΔH_m_c_ ^f^ [J·g^−1^]	T_c_ ^g^ [°C]	ΔH_c_ ^h^ [J·g^−1^]	T_g_ ^i^ [°C]	*χ_c_* ^j^ [%]
**Pure PLA prior to processing**	136.4	2.1	153.1	-	-	−30.4	-	-	58.2	32.6
**PLA after processing**	134.6	2.4	153.8	-	-	−16.1	127.5	13.5	59.4	2.7
**Exp PLA after processing**	89.8	2.4	150.5	−23.9	157.4	−37.6	119.9	37.1	58.4	0.6

^a^ weight average molecular weight; ^b^ molar mass dispersity; ^c^ melting temperature of first peak; ^d^ enthalpies of melting of first peak; ^e^ melting temperature of second peak; ^f^ total enthalpies of melting; ^g^ crystallization temperature; ^h^ enthalpies of crystallization; ^i^ glass transition temperature; ^j^ calculated crystallinity.

**Table 2:** Thermal properties of samples during abiotic hydrolysis experiments.

Sample	pH	Time (Week)	T_m_1_ ^a^ [°C]	ΔH_m_1_ ^b^ [J·g^−1^]	T_m_2_ ^c^ [°C]	ΔH_m_c_ ^d^ [J·g^−1^]	T_c_ ^e^ [°C]	ΔH_c_ ^f^ [J·g^−1^]	T_g_ ^g^ [°C]	*χ_c_* ^h^ [%]
**PLA**	2.4	0	153.8	-	-	−16.1	127.5	13.5	59.4	2.7
		4	153.4	-	-	−24.9	125.5	21.7	59.4	3.4
		12	152.4	-	-	−30.6	122.9	30.3	59.0	0.4
		20	150.7	−25.0	157.2	−37.0	119.9	36.1	57.9	1.0
**PLA**	7.4	0	153.8	-	-	−16.1	127.5	13.5	59.4	2.7
		4	153.6	-	-	−21.9	125.7	21.5	59.6	0.4
		12	152.5	-	-	−30.3	123.2	30.1	59.2	0.2
		20	150.7	−12.9	157.1	−37.1	117.9	35.6	58.5	1.6
**PLA**	12	0	153.8	-	-	−16.1	127.5	13.5	59.4	2.7
		4	153.2	-	-	−25.6	124.0	25.6	59.4	0.1
		12	153.4	-	-	−26.9	125.0	23.3	59.5	3.9
		20	151.6	−23.7	157.1	−33.0	121.4	32.5	58.7	0.5
**Exp PLA**	2.4	0	150.5	−23.9	157.4	−37.6	119.9	37.1	58.4	0.6
		4	155.8	−23.3	147.2	−42.6	114.1	38.4	57.1	4.5
		12	155.2	−24.2	145.9	−40.8	110.9	39.9	55.3	0.9
		20	153.8	−19.3	144.1	−30.0	108.7	30.0	54.0	0.0
**Exp PLA**	7.4	0	150.5	−23.9	157.4	−37.7	119.9	37.1	58.4	0.6
		4	150.4	−22.9	157.0	−36.5	120.4	35.2	57.9	1.4
		12	156.5	−18.9	148.5	−38.8	116.4	38.6	57.0	0.3
		20	156.2	−20.7	148.1	−39.7	115.2	37.5	56.9	2.4
**Exp PLA**	12	0	150.5	−23.9	157.4	−37.7	119.9	37.1	58.4	0.6
		4	156.9	−15.9	149.6	−37.6	118.2	35.8	58.0	1.7
		12	156.0	−18.9	148.1	−38.6	116.5	37.6	56.3	1.0
		20	155.0	−22.0	146.4	−39.7	113.9	38.0	55.3	1.9

^a^ melting temperature of first peak; ^b^ enthalpies of melting of first peak; ^c^ melting temperature of second peak; ^d^ total enthalpies of melting; ^e^ crystallization temperature; ^f^ enthalpies of crystallization; ^g^ glass transition temperature; ^h^ calculated crystallinity.
